# Decoding hand movement velocity from electroencephalogram signals during a drawing task

**DOI:** 10.1186/1475-925X-9-64

**Published:** 2010-10-28

**Authors:** Jun Lv, Yuanqing Li, Zhenghui Gu

**Affiliations:** 1 Center for Brain-Computer Interfaces and Brain Information Processing, College of Automation Science and Engineering, South China University of Technology, Wushan Road, Guangzhou, PR China

## Abstract

**Background:**

Decoding neural activities associated with limb movements is the key of motor prosthesis control. So far, most of these studies have been based on invasive approaches. Nevertheless, a few researchers have decoded kinematic parameters of single hand in non-invasive ways such as magnetoencephalogram (MEG) and electroencephalogram (EEG). Regarding these EEG studies, center-out reaching tasks have been employed. Yet whether hand velocity can be decoded using EEG recorded during a self-routed drawing task is unclear.

**Methods:**

Here we collected whole-scalp EEG data of five subjects during a sequential 4-directional drawing task, and employed spatial filtering algorithms to extract the amplitude and power features of EEG in multiple frequency bands. From these features, we reconstructed hand movement velocity by Kalman filtering and a smoothing algorithm.

**Results:**

The average Pearson correlation coefficients between the measured and the decoded velocities are 0.37 for the horizontal dimension and 0.24 for the vertical dimension. The channels on motor, posterior parietal and occipital areas are most involved for the decoding of hand velocity. By comparing the decoding performance of the features from different frequency bands, we found that not only slow potentials in 0.1-4 Hz band but also oscillatory rhythms in 24-28 Hz band may carry the information of hand velocity.

**Conclusions:**

These results provide another support to neural control of motor prosthesis based on EEG signals and proper decoding methods.

## Background

Brain-computer interface (BCI) is a system that translates brain signals reflecting user intentions into commands and drives external devices [1,2]. In the past decades, various BCI systems have been developed for the purpose of rehabilitation and medical care for the disabled patients [3-7]. Among them, researchers have particular interest in neuromotor prosthesis that moves an artificial limb by the brain signals which control the equivalent movement of a corresponding body part such as an arm or a hand [2]. To date, most progresses of these BCI systems have been based on invasive approaches using neuronal firing patterns [4,8,9], local field potentials (LFPs) [10,11] or electrocorticogram (ECoG) [12-14]. These signals inside head possess the advantages of little noise, high topographical resolution and broad bandwidth.

However, for applications on human being, invasive ways are seriously limited by questions about the safety and durability of implanted channels [15]. Some recent studies have demonstrated that brain signals recorded by non-invasive approaches also carry significant information of detailed limb movements. For instance, from magnetoencephalogram (MEG) signals, hand movement directions have been decoded in the discrete center-out reaching task [16]; hand positions have been decoded during the continuous joystick movements [17]; and hand velocities have been decoded during the discrete center-out drawing task [18], the target-to-target joystick movements [19] and the continuous trackball movements [20]. It has been reported that low frequency band (≤3 Hz or 2-5 Hz) MEG on motor-related areas is critically involved in representing limb movement direction and speed [16,20]. Moreover, long-distance coupling between primary motor cortex and multiple brain areas in the low frequency band has been found during a continuous visuomotor task [20]. And the neural mechanisms of speed and *tau *in pointing hand movement from MEG have been revealed (*tau *is defined as the ratio of the current distance-to-goal gap over the current instantaneous speed towards the goal) [19].

Compared with MEG, electroencephalogram (EEG) has lower signal-to-noise ratio and spatial resolution. It was generally thought that EEG could not extract sufficient information to reconstruct limb movements. However, EEG is easily available and more suitable for ambulatory prosthetic system [17,21]. Therefore, a few ambitious researchers have extended the exploration to EEG signals. For example, hand directions have been inferred from EEG recorded in a center-out joystick operation [16]. The subjects were constrained to small finger and wrist movements. Another study has been presented about the prediction of reaching target from EEG recorded in multi-joint center-out movements [22]. Later, a movement delay paradigm was designed to investigate brain activities in the human posterior parietal cortex (PPC) during the planning of intended movements [23]. Newly, the positions, the velocities and the accelerations of hand movement were modestly decoded during a 3-D center-out reaching task [24,25]. As far as we know, most of these EEG studies employed a center-out movement task which contained pre-specified point-to-point movements. Specifically, the starting and end points were fixed, and the length of each movement was well constrained.

**Figure 1:**
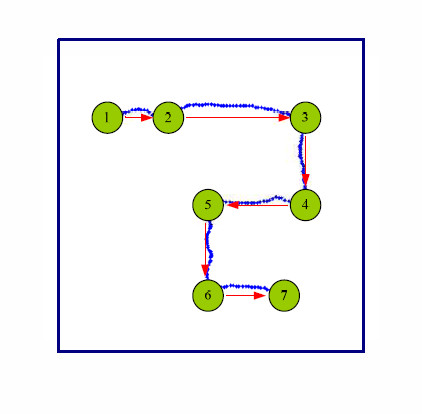
**Drawing task paradigm**. The example of movement trajectories (blue dotted lines) performed by a subject. Movement directions are displayed as the red arrows. The starting point is represented as green circle 1. It was randomly initialized by the laptop. The movement targets are denoted as circle 2 to circle 7. The number and positions of the targets were determined online by the subjects.

In our study, we designed a 2-D drawing task in which the subjects were required to move a pen at their own pace along a zigzag route in each trial (refer to Figure 1). This zigzag route was determined online by the subjects themselves. Specifically, this task can be regarded as sequential point-to-point movements. At each point the subjects selected one of the four directions, i.e., up, down, left and right. Moreover, the numbers and the positions of these points, and the distance between two sequential points were up to the subjects (not pre-specified). Thus the starting point, the end point and the length of each point-to-point movement were less restricted compared to the center-out task. During the experiment, multi-channel EEG activities from whole scalp were recorded. Then, independent component analysis (ICA) [26] was used to remove the effects of electrooculogram (EOG) and electromyogram (EMG) activities. After that, discriminative spatial pattern (DSP) filtering [27] and common spatial pattern (CSP) filtering [28] were employed to extract the amplitude features and the power features from the retained independent components (ICs) in multiple frequency bands. Then Kalman filtering and a smoothing algorithm [29] were applied to decode the hand movement velocity with these features. Furthermore, we investigated the scalp areas most involved for the decoding and evaluated the decoding performance of each frequency band.

## Methods

### Subjects and Recording System

Five right-handed healthy male subjects participated voluntarily in this study. Among them, subject 1 had been well trained in the BCI experiments of hand motor imageries, while the other subjects had less or never participated in any kind of BCI experiment before. These five subjects were instructed to move a pen (using their right wrist only and relaxing left hand on the lap) on the touch screen of a laptop in front of them. Meanwhile, the pen tracks denoting the trajectories of hand movements were recorded with a sampling rate of 64 Hz by the laptop. At the same time, a 40-channel EEG cap LT37 from Compumetics was used to collect EEG signals from the subjects. And a portable amplifier (NeuroScan NuAmps) amplified the analog EEG signals, digitalized them with a sampling rate of 250 Hz. The laptop received the EEG data from the amplifier through a USB port and sent synchronous stimulus code through parallel port to the amplifier.

### Experimental Paradigm

Our experiment contained 60 trials. Each trial started with a fixation cross shown on the touch screen for 2 seconds. After that, a graphical user interface (GUI) was displayed. It was a 7 cm × 7 cm square in which a green ball denoting the starting point was randomly initialized. Then, in the next 40-50 seconds, subjects were asked to touch the green ball by a pen and move it to arbitrary points at their own pace in 4 directions (up, down, left and right). An example of the task is shown in Figure1. Actually, the pen track of each trial corresponded to a sequence of directional hand movements. In this experiment, the subjects self-chose the number of point-to-point movements during the drawing task. After the drawing time slot, this GUI disappeared and the trial was ended. The time for rest between the trials was randomized in a range from 8 s to 10 s to prevent subjects getting used to the timing of rest state to drawing task. During the time for rest, subjects were periodically told which directions were under-represented by the laptop for data balance. More detailed parameters of this experiment are listed in Table 1.

**Table 1:** The detailed parameters of the drawing task

	S1	S2	S3	S4	S5
TR(mm/s)	3.7 ± 2.1	11.3 ± 7.5	7.2 ± 3.2	5.9 ± 3.8	4.6 ± 2.2
DT_R(s)	10.9 ± 2.9	8.0 ± 1.9	9.4 ± 2.7	9.1 ± 3.1	9.4 ± 2.8
DT_U(s)	12.0 ± 2.4	8.6 ± 2.5	10.3 ± 2.5	12.0 ± 4.1	11.4 ± 3.6
DT_L(s)	11.9 ± 2.7	8.2 ± 1.8	8.2 ± 2.4	9.9 ± 3.6	10.2 ± 3.0
DT_D(s)	11.6 ± 2.8	8.7 ± 1.7	12.8 ± 2.3	11.4 ± 3.1	10.4 ± 3.3
MT(s)	8.2 ± 4.2	2.2 ± 1.5	4.6 ± 2.9	4.2 ± 3.7	7.3 ± 3.4
ML(mm)	31.6 ± 15.8	24.5 ± 17.6	33.3 ± 20.2	24.8 ± 21.0	33.8 ± 15.7

The mean ± standard deviation of the experiment parameters are shown for each subject. The abbreviations of these parameters are listed below:

(1) TR: subject's drawing speed during the entire time period of an experiment;

(2) DT_R: movement time in 'right' direction in a trial;

(3) DT_U: movement time in 'up' direction in a trial;

(4) DT_L: movement time in 'left' direction in a trial;

(5) DT_D: movement time in 'down' direction in a trial;

(6) MT: the time of a point-to-point movement;

(7) ML: the distance of a point-to-point movement.

### EOG and EMG removal

During our drawing tasks, the recorded EEG signals were contaminated with various artifacts such as EOG and EMG [30]. These artifacts may confound the EEG decoding of hand movements [18]. To show an example, we collected the EOG of Subject 3 and provided an off-line analysis in Appendix A1. The off-line analysis of EOG and the decoding of hand velocity of Subject 3 were based on the same dataset. To remove EOG and EMG, we employed ICA. It is a process that detects and isolates independent components (ICs) of signals consisting of mixed sources. For each subject, 30 ICs were decomposed from EEG signals by using the EEGLAB software [31], and about 12 ICs regarded as EOG/EMG were removed by the following heuristics: (i) Eye movements should project mainly to frontal sites with a low-pass time course; (ii) Eye blinks should project to frontal sites and have large punctate activations; (iii) Temporal muscle activities should project to temporal sites with a spectral peak in the band above 20 Hz [32]. An example of EOG and EMG removal is also given in Appendix A1.

### Feature extraction

Since the direction was approximately fixed (up, down, left or right) in each point-to-point movement in our study, the values of hand velocities have close relationship with the directions. For example, when a subject performed a movement to the right, the absolute value of hand velocity in y-dimension is small and the hand velocity in x-dimension is large. It may suggest that the brain components discriminative for different directional movements were helpful for reconstructing the profiles of hand velocities. Therefore, supervised spatial filtering methods CSP and DSP were employed here to extract the discriminative brain components. Specifically, after EOG and EMG were removed, a filter bank was applied to filter the retained ICs into multiple bands (0.1-4 Hz, 4-8 Hz, 8-12 Hz, ..., 36-40 Hz). Then DSP was used to extract the amplitude features of slow potentials within 0.1-4 Hz band of the ICs. And CSP was applied to extract the power features of oscillatory rhythms from the other bands of the ICs. The details of DSP and CSP methods can be found in Appendix A2.

In DSP and CSP training procedure, we cut hand movement trajectories into segments with a sliding window (1s wide and 0.5s overlap) to obtain the directions in the drawing task. It was expected that the trajectory in each segment only exhibits one movement direction. However, in practice, the trajectories of some segments may not be straight lines or not extend enough in a direction. The ICs of these segments were not used into DSP or CSP training. Note that DSP and CSP were originally proposed to deal with binary classification problems. As far as our 4-direction hand movements are concerned, DSP and CSP need to be extended to multiclass paradigms. In this study, they were computed between each pair of directions [33], and the number of the pairs was $C_{4}^{2}=6$.

After CSP/DSP filter training was completed, regarding each pair of directions, 2 most discriminating filters of DSP and 4 most discriminating filters of CSP were obtained (see Appendix A2). Then they were used to filter the multi-band ICs into time series. In each frequency band, the combination of ICA and DSP/CSP can be formulated as:

(1) $$\xi_{i}=W_{i}^{\text{T}}(UX_{i})$$

where **X**_*i *_∈ *R*^*C*×*T *^is the recorded EEG signal in the *i*th frequency band, *i *= 1,2,...,10, *C *is the number of channels, *T *is the number of sample points covering the entire time period of an experiment, **U **∈ *R*^*m*×*C *^is the 'unmixing' matrix of ICA, *m *is the number of retained ICs, $W_{i}\in R^{m\times l_{i}}$ is the filtering matrix of DSP or CSP in the *i*th frequency band, *l*_*i *_is the number of the selected filters (*l*_1 _= 12, *l*_2 _= *l*_3 _=...= *l*_10 _= 24), $\xi_{i}\in R^{l_{i}\times T}$ is the filtered data.

At last, we extracted the features from the filtered data **ξ**_*i *_every 200 ms without overlap, i.e., **ξ**_*i *_= [**ψ**_*i*1_, **ψ**_*i*2_,..., **ψ**_*iN*_], where *N *is the number of 200 ms bins. Within each 200 ms bin, the average amplitudes of 0.1-4 Hz signals were calculated as $z_{1,j}(q)=means(\psi_{1,j}^{q})$, where $\psi_{1,j}^{q}$ is the *q*th row of **ψ**_1,*j*_, *j *= 1,2,..., *N*, *q *= 1,2,...,12. The variances of the other frequency band signals were computed, normalized and log-transformed as $z_{i,j}(p)=\log\left\{\mathrm{var}(\psi_{i,j}^{p})/\sum_{p=1}^{24}\mathrm{var}(\psi_{i,j}^{p})\right\}$, where $\psi_{i,j}^{p}$ is the *p*th row of **ψ**_1,*j*_, *i *= 2,3,...,10, *p *= 1,2,...,24. Before decoding, these features were normalized to zero mean and unit variance. They were denoted as **z**_*j *_= [*z*_1,*j*_, *z*_2,*j*_, ..., *z*_10,*j*_], **z **∈ *R*^*D*×*N*^, where *D *= 228 is the dimension of features. Moreover, in this paper, x-velocity and y-velocity of the hand movement were measured as the displacements of pen track on horizontal dimension and vertical dimension within each 200 ms bin, respectively.

### Decoding Algorithms

The decoding algorithm presented in this paper consists of a standard Kalman filter and a smoother. The Kalman filter is a real-time processing algorithm in which the state estimate is updated immediately after a new observation is available. On the other hand, the smoother optimally combines the Kalman filter with a reverse-time information filter. The result is a minimum variance estimate based on past, present and future information [34].

#### (1) Kalman filter

Kalman filter considers a discrete filtering model [29], of which the system and observation models are:

(2) $$v_{j+1}=A_{j}v_{j}+n_{j}$$

(3) $$z_{j}=H_{j}v_{j}+q_{j}$$

In this paper, the state vector is denoted by **v**_*j *_= [*v*_*x, j*_, *v*_*y, j*_]^T ^with *v*_*x, j *_and *v*_*y, j *_representing the horizontal and the vertical velocities respectively at time step *j*; **A**_*j *_∈ *R*^2×2 ^is the state transition matrix, and **n**_*j *_~ *N *(0,**N**_*j*_) is the noise term, where **N**_*j *_∈ *R*^2×2^. The observation vector **z**_*j *_∈ *R*^*D *^is made up of the extracted features, **H**_*j *_∈ *R*^*D*×2 ^is the observation matrix, and **q**_*j *_~ *N*(0,**Q**_*j*_) is the noise term of observation, where **Q**_*j *_∈ *R*^*D*×*D*^, *D *= 228, *j *= 1,2,..., *M*_*k*_, and *M*_*k *_is the number of time steps in the *k*th trial. Here **A**_*j*_, **H**_*j*_, **N**_*j *_and **Q**_*j *_are simplified as constant matrices. The matrices **A **and **H **can be obtained from training data by using least squares estimation:

$$\begin{array}{c} \underset{A}{\arg\min}\sum_{k\in Tr}\sum_{j=1}^{M_{k}-1}{\left\|v_{k,j+1}-Av_{k,j}\right\|}^{2}\\ \underset{H}{\arg\min}\sum_{k\in Tr}\sum_{j=1}^{M_{k}}{\left\|z_{k,j}-Hv_{k,j}\right\|}^{2} \end{array}$$

where *Tr *is the set of training trials. For the estimated **A **and **H**, the noise covariance matrices **N **and **Q **can be obtained by equation (2) and (3). The prediction and update equations of Kalman filter for test can be written as follows [29]:

$$\begin{array}{ll} Prediction: & {\hat{v}}_{j}^{-}=A{\hat{v}}_{j-1}\\ & P_{j}^{-}=AP_{j-1}A^{\text{T}}+N \end{array}$$

$$\begin{array}{ll} Update: & S_{j}=HP_{j}^{-}H^{T}+Q\\ & K_{j}=P_{j}^{-}H^{\text{T}}S_{j}^{-1}\\ & {\hat{v}}_{j}={\hat{v}}_{j}^{-}+K_{j}(z_{j}-H{\hat{v}}_{j}^{-})\\ & P_{j}=P_{j}^{-}-K_{j}S_{j}K_{j}^{\text{T}} \end{array}$$

where ${\hat{v}}_{j}^{-}$ and $P_{j}^{-}$ are the predicted mean and covariance of the state before seeing **z**_*j*_; **S**_*j *_is the prediction covariance of the observation; ${\hat{v}}_{j}$ and **P**_*j *_are the estimated mean and covariance of the state after seeing **z**_*j*_, **K**_*j *_is the filter gain.

#### (1) Smoother

The smoother is calculated from the results of Kalman filter by recursions [34]:

$$\begin{array}{l} C_{j}=P_{j}A_{}^{\text{T}}{\left[AP_{j}A_{}^{\text{T}}+N\right]}^{-1}\\ {\hat{v}}_{j}^{s}={\hat{v}}_{j}+C_{j}[{\hat{v}}_{j+1}^{s}-A{\hat{v}}_{j}]\\ P_{j}^{s}=P_{j}+C_{j}[P_{j+1}^{s}-AP_{j}A_{}^{\text{T}}-N]C_{j}^{\text{T}} \end{array}$$

where **C**_*j *_is the smoother gain; ${\hat{v}}_{j}$ and **P**_*j *_are the filter estimates for the state mean and state covariance; ${\hat{v}}_{j}^{s}$ and $P_{j}^{s}$ are the smoother estimates for the state mean and state covariance. The recursions start from the last time step.

## Results

To study the fidelity of the drawing movement decoding and the characteristics of the associated EEG signals, we will show the accuracy of the hand velocity decoding, demonstrate the scalp areas most involved for the decoding and present the frequency bands that carried information of hand velocity. 5-fold cross-validation was employed in the evaluation, i.e., each subject's data were divided into 5 parts, among them 4 parts were used for training, and the retained part was adopted for test. This procedure was repeated 5 times. In each time, a different part was used as the test set. The results of these evaluations are described below.

### Decoding accuracy of drawing movement

Table 2 shows three performance indexes to assess the decoding accuracy, including (i) Pearson correlation coefficient (*r*-value), abbreviated as CC, between the measured and the decoded hand velocities; (ii) *p*-value for testing the null hypothesis that the measured and the decoded hand velocities are uncorrelated by Student's *t*-test; (iii) signal-to-noise ratio (SNR), where $SNR=10\log_{10}[(E(v^{2})/E{\left(v-\hat{v}\right)}^{2}]$, *v *denotes the measured hand velocity, $\hat{v}$ represents the decoded hand velocity.

**Table 2:** Decoding performance of hand velocity using ICA-cleaned EEG

	S1	S2	S3	S4	S5	Avg.
CC_x_	0.62 ± 0.05	0.29 ± 0.03	0.50 ± 0.03	0.29 ± 0.03	0.16 ± 0.01	0.37 ± 0.08
CC_y_	0.04 ± 0.02	0.17 ± 0.02	0.39 ± 0.03	0.28 ± 0.03	0.30 ± 0.02	0.24 ± 0.06
						
*p*_x_	0	0	0	0	1.84 × 10^-9^	-
*p*_y_	0.08	1.17 × 10^-7^	0	0	0	-
						
SNR_x_(dB)	2.14 ± 0.41	0.30 ± 0.12	1.19 ± 0.13	0.35 ± 0.08	0.09 ± 0.02	0.81 ± 0.38
SNR_y_(dB)	-0.06 ± 0.03	0.05 ± 0.08	0.66 ± 0.09	0.34 ± 0.06	0.36 ± 0.04	0.27 ± 0.13

Pearson correlation coefficients (CCs), *p*-values and signal-to-noise ratios (SNRs) between measured and decoded hand velocities in x-dimension and y-dimension are listed. Top group: the mean ± stand error of the mean (SEM) of CCs is given for each subject and dimension across all 5 folds. The average of CCs across subjects is also given. Middle group: the mean of *p*-values is provided for each subject and dimension across all 5 folds. Button group: the SNRs are recorded for each subject and dimension across all 5 folds. The average of SNRs across subjects is also given. Before computing CC, *p*-value and SNR, the measured and decoded hand velocities were smoothed with a zero-phase, fourth-order, lowpass Butterworth filter with a cut-off frequency of 1 Hz.

From Table 2, we can find that, except the result of Subject 1 in y-dimension, the small *p*-values indicate that the CCs are significant. On average, the modest CCs and SNRs demonstrate that it is possible to infer information about hand velocities in drawing task by EEG. For most subjects, the hand velocities in horizontal dimension, x, were better decoded than those in vertical dimension, y. Similar disparity in the MEG decoding between dimensions of hand movement has been discussed in [35]. Because the subjects were asked to draw on the vertical touch screen, gravitational force may impact the drawing action of subjects and degrade the decoding in y-dimension [35]. Although we only presented the results for one parameter setting (1s segment length for CSP/DSP filter training and 200 ms step size for Kalman smoother decoding), it was also found that these parameters could be chosen in a wide range. For instance, we also tried other parameter settings (segment length for CSP/DSP filter training: 0.5s and 2s; decoding step size: 100 ms and 300 ms), and obtained comparable results. These results are not included in this paper due to limited page space.

**Figure 2:**
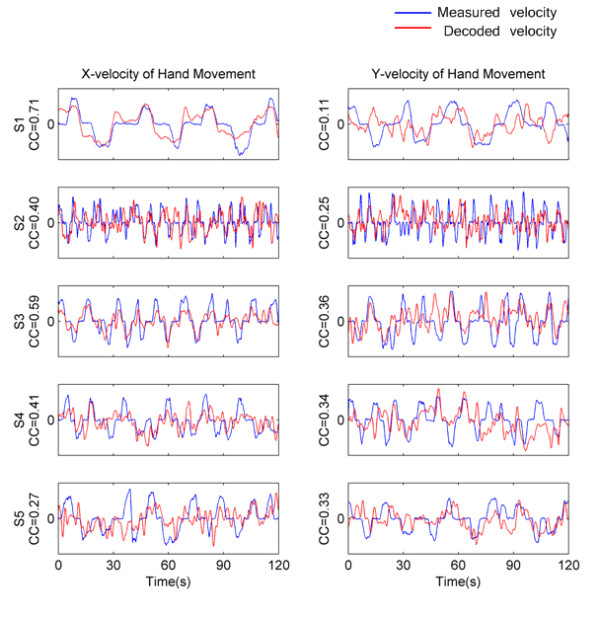
**Decoding examples**. Examples of smoothed and standardized measured (blue) and decoded (red) hand velocities. The left column is for x-dimension, and the right column is for simultaneous y-dimension. Each row contains data for one subject. The Pearson correlation coefficient (CC) between measured and decoded velocities is listed for each subplot.

Some examples of measured and decoded hand velocities in x-dimension and y-dimension are displayed in Figure 2. It can be seen that, in y-dimension, the decoded velocities hardly reflect the trends of the measured ones, while in x-dimension, generally, the decoded velocities match the measured ones better. Meanwhile, the measured velocities roughly consist of sequential bell shapes. Each bell shape indicates a relative straight trajectory made by a subject in a certain direction. Note that most bell shapes are irregular, which may be caused by two facts (i) the variable friction exists between the pen and the touch screen; (ii) visual guided point-to-point movements are not implemented in a purely feed-forward manner [19].

### Scalp areas most involved for hand velocity decoding

**Figure 3:**
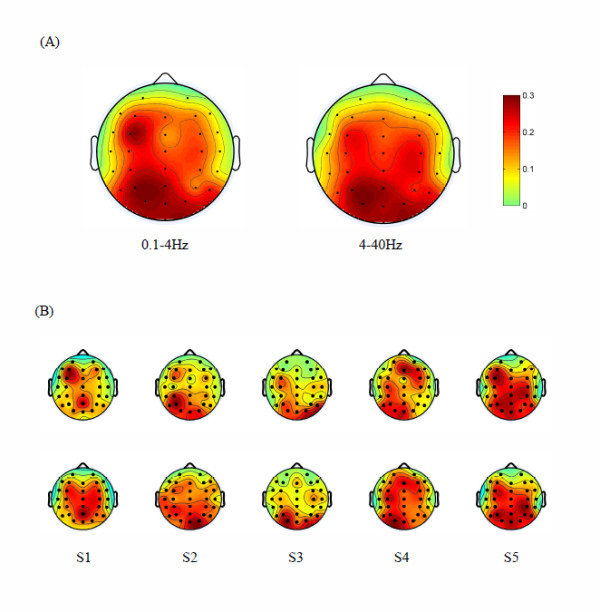
**Scalp topographies of channel weights according to the feature extraction for velocity decoding**. (A) This figure shows the averaged scalp topographies of channel weights across five subjects in 0.1-4 Hz (left) and 4-40 Hz (right), respectively; (B) This figure shows the scalp topographies of channel weights for the five subjects in 0.1-4 Hz (upper row) and 4-40 Hz (lower row), respectively.

Note that the brain components were generated by applying ICA and CSP/DSP to EEG signals. We rewrite equation (1) as

$$\xi_{i}=B_{i}X_{i}$$

where $B_{i}=W_{i}^{\text{T}}U$, $B_{i}\in R^{l_{i}\times C}$, *l*_*i *_is the number of selected filters in the *i*th frequency band, *i *= 1,2,...,10, *C *is the number of channels. Each row of **B**_*i *_gives a weight vector for channels to construct a brain component. Regarding velocity decoding by Kalman model, the observation is consisted of the features extracted from these brain components. Thus **B**_*i *_partly reflects the importance of the channels for velocity decoding. To investigate which channels were more involved for the velocity decoding in the *i*th frequency band, we average the rows of **B**_*i *_as follows:

$$I_{i}=\frac{1}{l_{i}}\sum_{q=1}^{l_{i}}\left|B_{i}^{q}\right|$$

where $B_{i}^{q}$ is the *q*th row of **B**_*i*_, $B_{i}^{q},I_{i}\in R^{C}$, |·| is an element-wise absolute operator. Figure 3 shows the scalp topographies of **I**_1 _and ($\sum_{i=2}^{10}I_{i}/9$) corresponding to the frequency bands 0.1-4 Hz and 4-40 Hz respectively.

Figure 3(A) presents the average scalp topographies across the 5 subjects. Generally, the contralateral and ipsilateral channels in motor, posterior parietal and occipital areas have greater weights, and the contralateral dominance is demonstrated. Specifically, for amplitude features in low frequency band (0.1-4 Hz), the channels over premotor, posterior parietal and occipital areas get greater weights; for power features in 4-40 Hz, the channels over posterior parietal and occipital areas get greater weights. These findings suggest the widespread involvement of brain areas with hand kinematics during the drawing task. The results are approximately in accordance with the following studies: Wang et al. demonstrated that intended movement directions can be predicted by recording EEG from posterior parietal areas [23]; Bradberry et al. showed that the sensorimotor area is important for hand velocity decoding [24]; And Vaillancourt DE et al. presented that the parietal and premotor cortex are associated with visuomotor processes [36].

Figure 3(B) displays the scalp topographies separately for each subject. On the whole, the channels on motor, posterior parietal and occipital areas get greater weights both in 0.1-4 Hz band and in 4-40 Hz band for all the subjects, although the weights of these areas are subject-dependent. As an exception, for Subject 4, the channels on prefrontal area also get greater weights. It may have been caused by some artifacts.

### Decoding performance of different frequency bands

**Figure 4:**
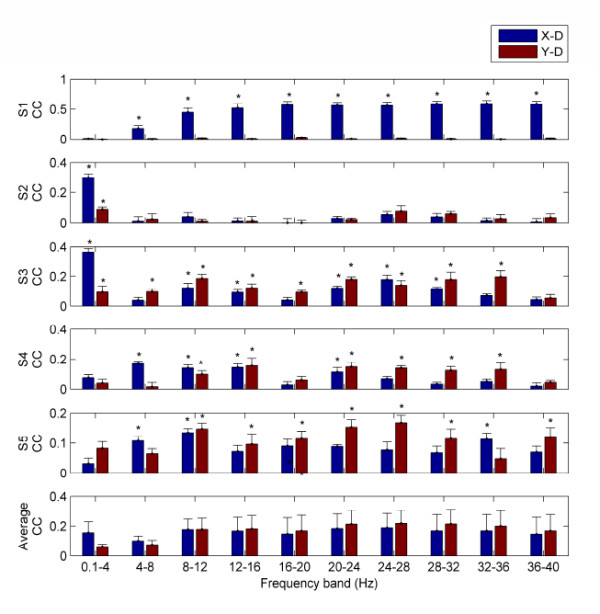
**Decoding performance of different bands**. By using the features from different frequency bands respectively, we show the mean and SEM of the Pearson correlation coefficients (CCs) between measured and decoded hand velocities across cross-validation folds for each subject in x-dimension (blue) and y-dimension (red). Stars indicate the bars with significant CCs (*p *< 0.05 for no correlation hypothesis, Student's *t*-test). The average CCs across subjects for each band feature are also given.

In order to explore which frequency bands carry information about hand velocity, we studied the decoding performance of each band, and show them in Figure 4. It can be seen that the frequency distribution for decoding is highly subject-dependent. For example, for Subject 1, the CC value of low frequency band (0.1-4 Hz) is significantly inferior to those of the other frequency bands in x-dimension (*p *< 0.05, paired left-tailed Student's t-test). However, for Subject 3, the CC value of low frequency band (0.1-4 Hz) is significantly superior to those of the other frequency bands in x-dimension (*p *< 0.05, paired right-tailed Student's t-test). Moreover, the CC values for Subject 1 are essentially zero in y-dimension for all the frequency bands and about 0.5 in x-dimension above 8 Hz. This may be due to the following fact. Subject 1 has been well trained for cursor control in a BCI system through left and right hand movement imageries. His voluntary power modulation of 8-40 Hz rhythms has been reinforced. The drawing task performed by right hand may have activated this power modulation in x-dimension which masks the information about hand movement in y-dimension. For Subject 2, the poor CC values of the frequency bands beyond 4 Hz indicated that, for certain people, the information about limb kinematics may not be inferred from the EEG above 4 Hz. The study of Waldert et al. provided similar results [16]. Regarding the average across all the subjects, there is no significant difference between the CC values of the low frequency band (0.1-4 Hz) and those of the other frequency bands in x-dimension (*p *> 0.40, paired two-tailed Student's t-test); however, the CC values of the frequency band from 24 Hz to 28 Hz are significantly higher than those of the low frequency band (0.1-4 Hz) in y-dimension (*p *< 0.05, paired right-tailed Student's t-test). These findings imply that, besides the slow potentials from 0.1 Hz to 4 Hz, the oscillatory rhythms from 24 Hz to 28 Hz may also carry notable information about hand movement velocity.

### Comparison on decoding performance with ICA-cleaned data and non-ICA-cleaned data

Here we list the decoding performance (CC) with non-ICA-cleaned data in Table 3. Comparing the CCs in Table 2 and Table 3, we can find that non-ICA-cleaned data result in remarkably higher decoding accuracies in x-dimension and in y-dimension (*p *< 0.05, paired right-tailed Student's t-test). It indicates that the components removed by ICA could offer considerable contribution to hand velocity decoding. Although most of these components are EOG and EMG (see Appendix A1), these removed components may contain EEG signals which carry the information of hand velocity to some degree.

**Table 3:** Decoding performance of hand velocity using non-ICA-cleaned EEG

	S1	S2	S3	S4	S5	Avg.
CC_x_	0.62 ± 0.05	0.35 ± 0.02	0.51 ± 0.03	0.49 ± 0.02	0.30 ± 0.03	0.46 ± 0.06
CC_y_	0.07 ± 0.03	0.22 ± 0.03	0.46 ± 0.03	0.38 ± 0.02	0.35 ± 0.05	0.30 ± 0.07

This table shows the mean ± SEM of CCs between measured and decoded hand movement velocities across five subjects based on non-ICA-cleaned data, in horizontal and vertical dimension respectively.

### Comparison on decoding performance of linear filter, Kalman filter and Kalman smoother

**Figure 5:**
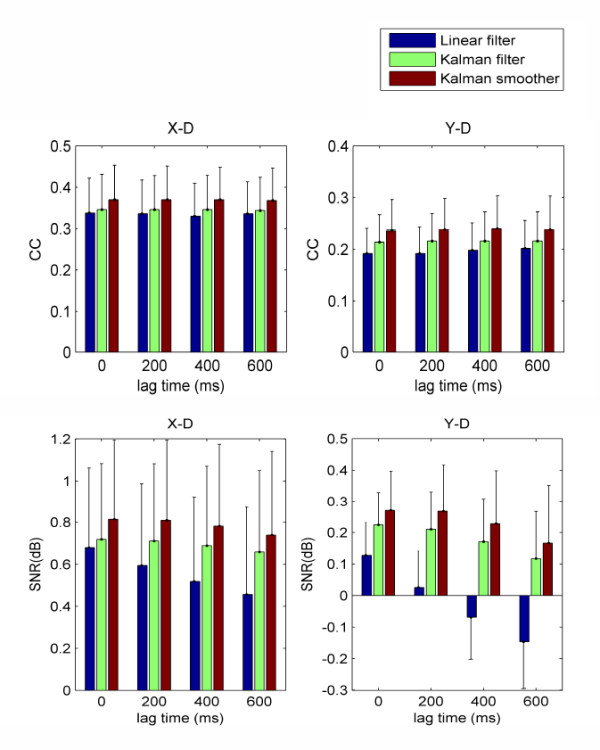
**Comparison on decoding performance of linear filter, Kalman filter and Kalman smoother**. This figure shows the mean (bar) with SEM (error bar) of CC (the first row) and SNR (the second row) across the 5 subjects with different lag time using linear filter, Kalman filter and Kalman smoother. In the calculation of SNR, decoding error and measured hand velocity are considered as noise and signal respectively.

Until now, many decoding algorithms have been used to reconstruct hand velocities, such as linear filter in the study of Bradberry et al. [24] and Kalman filter in the research of Wu et al. [37]. As discussed in [37], compared with linear filter, Kalman filter possesses the advantages of a clear probabilistic foundation and a model of the temporal hand kinematics. Based on the work of Wu et al. [37], here we employed the smoothing method to integrate not only past and present information but also future information of hand velocities into Kalman model. With different lag time, the average decoding performance across five subjects for linear filter, Kalman filter and Kalman smoother are shown in Figure 5. Paired Student's t-test is employed to compare the decoding performance of the three methods. The results are listed in Table 4. From Figure 5 and Table 4, we find that with different lag times, the CCs and SNRs of Kalman smoother are significantly better than those of the linear filter and Kalman filter (*p *< 0.05, right-tailed), except in y-dimension where the SNRs of Kalman smoother are not significantly superior to those of Kalman filter (*p *> 0.05, right-tailed). Considering the Kalman smoother in this paper being an off-line algorithm, we plan to modify it and extrapolate this work to an online system in the future.

**Table 4:** Comparison on decoding performance of Kalman smoother and the other methods

	Lag = 0 ms	Lag = 200 ms	Lag = 400 ms	Lag = 600 ms
Kalman smoother	X-D: *p *= 0.0163	X-D: *p *= 0.0163	X-D: *p *= 0.0209	X-D: *p *= 0.0163
vs. Kalman filter	Y-D: *p *= 0.0257	Y-D: *p *= 0.0257	Y-D: *p *= 0.0120	Y-D: *p *= 0.0314
Kalman smoother	X-D: *p *= 0.0061	X-D: *p *= 0.0037	X-D: *p *= 0.0024	X-D: *p *= 0.0027
vs. Linear filter	Y-D: *p *= 0.0122	Y-D: *p *= 0.0074	Y-D: *p *= 0.0098	Y-D: *p *= 0.0163
				
Kalman smoother	X-D: *p *= 0.0107	X-D: *p *= 0.0096	X-D: *p *= 0.0133	X-D: *p *= 0.0258
vs. Kalman filter	Y-D: *p *= 0.0542	Y-D: *p *= 0.0544	Y-D: *p *= 0.0791	Y-D: *p *= 0.1230
Kalman smoother	X-D: *p *= 0.0022	X-D: *p *= 0.0018	X-D: *p *= 0.0012	X-D: *p *= 0.0007
vs. Linear filter	Y-D: *p *= 0.0034	Y-D: *p *= 0.0017	Y-D: *p *= 0.0013	Y-D: *p *= 0.0011

Top group: comparison on CCs using paired right-tailed Student's t-test. Button group: comparison on SNRs using paired right-tailed Student's t-test.

## Discussion

### Comparison with other related studies

In this paper, the average CC across the five subjects over x-dimension and y-dimension is 0.30. As the most related work, hand velocity was reconstructed from EEG during a 3-D center-out reaching task, and a very close CC (0.29) was obtained [24]. In addition, MEG signals also reflect the activities of large neuronal populations. From MEG, hand velocities were predicted during a 2-D center-out drawing task, and a higher CC (0.4) was gained without EOG or EMG removal [18]. Therefore, the decoding accuracy of our work is within the range of those achieved in the studies mentioned above. Moreover, we would like to compare the experimental paradigms in this paper and that in [24] as below:

(i) In [24], the center-out task is a 3D reaching movement, in which the subject moved his hand from a fixed starting point (center) to one of the 8 stationary targets, and then moved his hand back to the center. In this paper, the task is a 2D self-routed drawing movement, in which the subject was required to move a pen at his own pace along a zigzag route in each trial. This task can be regarded as sequential point-to-point movements. At each point the subject selected one of the four directions. Moreover, the numbers and positions of these points, and the distance between two sequential points were up to the subject. Therefore, compared to [24], the starting point, the end point and the length of each point-to-point movement in our experiments were less constrained. The subjects can perform the movements with higher variability. It has been reported in [24] that the variabilities of movement time and movement length are negatively correlated with the accuracy of hand velocity decoding. From this viewpoint, the hand velocity of our drawing movement could be harder to decode than that of the center-out movement task.

(ii) In [24], subjects were asked to perform multi-joint movements of the upper limb. In our work, the subjects were instructed to make movements only with their hands and wrists, while keeping their shoulders and arms at rest. We studied hand movements not only because of the interesting work on hand movement direction decoding [16], but also because hand is relatively far from the EEG cap, therefore reduces EMG contamination to the EEG signals. Since our drawing task needs the coordination of eye and hand, EOG and EMG may confound the EEG decoding. Thus we employed ICA to remove EOG and EMG artifacts.

### Decoding hand kinematics in different frequency bands

Which frequency band of neural signal carries most information about limb kinematics is an important issue discussed in the existing studies. For example, Ball et al. summarized the decoding accuracies of arm movement direction with different band ECoG, and indicated that highest decoding accuracy can be obtained from slow movement-related potentials (MRPs) (<2 Hz) [38]. Jerbi et al. reported the notable phase locking between 2-5 Hz MEG oscillatory activity in the contralateral primary motor cortex and time-varying hand speed [20]. Regarding EEG recording, Waldert et al. discovered that low frequency band (≤3 Hz) EEG of the sensors located in the motor-related area have close relationship with movement directions [16]. In addition, it is well known that the planning and execution of movement leads to significant power modulation in 8-30 Hz EEG, i.e., event-related synchronization/desynchroniza- tion (ERS/ERD) [39,40]. Such characteristic changes in EEG rhythms have been used to classify brain states related to the planning/imagery of different types of limb movement [41]. Newly, Han et al. reported that EEG activities in the alpha (8-12 Hz) and beta (18-28 Hz) frequency bands were correlated with the speed of imagery clenching [42]. In our study, we have shown that displacement velocity can be represented by the MRP in 0.1-4 Hz band and the ERD/ERS in 24-28 Hz band. Further more, we analyzed the relevance of decoding results from different frequency bands (see Appendix A3), and found that the decoding results of MRPs from low frequency band (0.1-4 Hz) are little correlated with those of oscillation rhythms from higher frequency bands (4-40 Hz). It indicates that the potential shifts in the low frequency band and the power modulations in the higher frequency bands reflect different aspects of brain activities related to hand movement velocity. Furthermore, from the scalp map in Figure 3 (A), we find that in the low frequency band, the channels in the motor, posterior parietal and occipital areas get greater weights. This demonstrates that the features in the low frequency bands capture the neural signature. The finding is in accordance with the ECoG study of Schalk et al. which also focused on decoding kinematic parameters of hand movement [14].

## Conclusions

Decoding limb kinematics from brain signals in non-invasive ways may realize safe and convenient control of motor prosthesis. In this paper, we demonstrated that EEG signals can be used to decode hand velocity during a sequential drawing task. The scalp areas over motor cortex, posterior parietal cortex and occipital areas were most involved for the decoding. Furthermore, we show that not only slow potentials in 0.1-4 Hz band, but also oscillatory rhythms in 24-28 Hz band may carry information about hand velocity.

## Competing interests

The authors declare that they have no competing interests.

## Authors' contributions

JL participated in the design of the study, carried out the experiment and data analysis, and drafted the manuscript. YL conceived of the study, and participated in its design and coordination. JL, YL and ZG read and approved the final manuscript.

## Appendix

### A1. EOG and EMG removal based on ICA

In our study, we recorded Subject 3's EOG activity with a bipolar sensor montage with sensors attached superior and inferior to the orbital fossa of the right eye for vertical eye movements and to the external canthi for horizontal eye movements. Firstly, we computed Pearson correlation coefficient (CC) and *p*-value (for no correlation hypothesis, Student's t-test) between the EOG signal and the measured hand velocity. The results are listed in Table 5. It is found that the correlation between the horizontal EOG activity and the horizontal hand velocity is significant (*p *< 0.001).

**Table 5:** Correlation between EOG activity and hand velocity

	Vertical EOG	Horizontal EOG
Horizontal hand velocity	CC = 0.04 (*p *= 0.16)	CC = 0.14 (*p *= 1.12 × 10^-6^)
Vertical hand velocity	CC = 0.01 (*p *= 0.73)	CC = 0.03 (*p *= 0.30)

This table shows the CCs and *p*-values between EOG activity and hand velocity covering the entire time period of an experiment, in horizontal and vertical dimension respectively.

**Figure 6:**
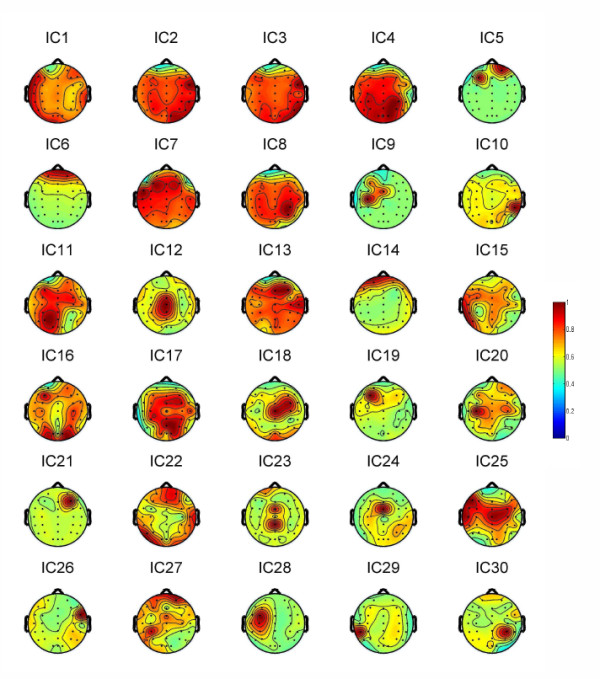
**Regularized Scalp maps of all the independent components (ICs)**. This figure shows the scalp maps of all the ICs based on the data of Subject 3.

Next, we removed EOG and EMG artifacts using ICA method. ICA removes artifacts from EEG records by eliminating the contributions of artifact sources to the scalp sensors. Using the data from Subject 3, we provided the regularized scalp maps of all the ICs in Figure 6.

**Figure 7:**
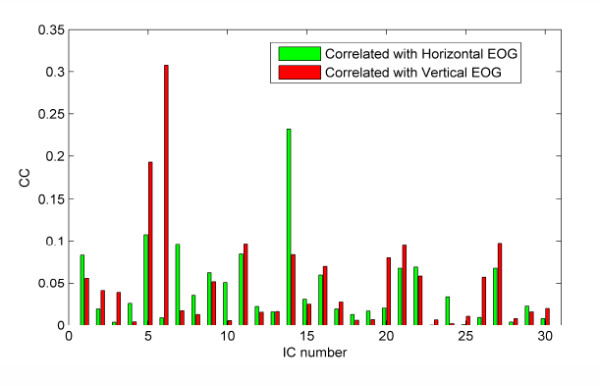
**Correlation coefficients between EOG activities and independent components (ICs)**. This figure shows the correlation coefficients (CCs) between the ICs and EOG in horizontal and vertical direction respectively.

From Figure 6, we can find that the projection strengths of IC5, IC6 and IC14 were concentrated on Fp1 or Fp2. These ICs were removed as the eye movement artifacts [31]. To demonstrate the validity of ICA for EOG removal in our study, we have computed the CCs between the independent components (ICs) and the recorded EOG activities. The results are shown in Figure 7, where we can observe that, except IC5, IC6 and IC14, all the components are not obviously correlated with EOG activities.

**Figure 8:**
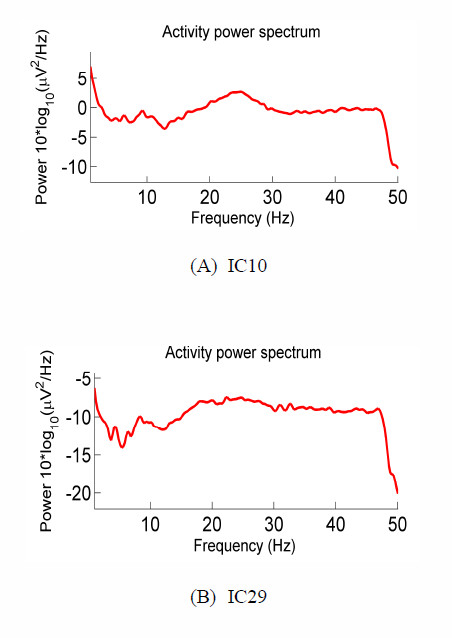
**Power spectrums of EMG independent components**. This figure shows the power spectrums of IC10 (A) and IC29 (B). The corresponding scalp maps are shown in Figure 6.

On the other hand, from Figure 6, we can find the projection strengths of IC10 and IC29 are concentrated on the temporal sites. Their power spectrums are shown in Figure 8, which demonstrates high power at frequencies above 20 Hz. Here, IC10, IC29 were removed as the EMG artifacts [31]. In our study, some ICs partially exhibit the characters of EOG/EMG, such as IC1, IC7, IC13, IC15, IC21, IC22, IC25, IC26 and IC27. They were also removed.

### A2. Details of DSP and CSP algorithms

Both DSP and CSP are linear projection methods [27,28]. They have the same data model as **Y **= **W**^T ^**X **, where **Y **∈ *R*^*C*×*T *^denotes the source component, **W **∈ *R*^*C*×*C *^is the projection matrix and **X **∈ *R*^*C*×*T *^represents the EEG segment, with *C *denoting the number of channels, and *T *denoting the number of samples in the time interval of interest.

However, the goals of DSP and CSP are different. For DSP, **W **is sought for the purpose of extracting the amplitude of slow non-oscillatory source. It projects EEG segments to the linear subspace where the between-class separation is maximized while the within-class separation is minimized. The projection vector achieving the largest ratio of between-class separation and within-class separation is defined as the most discriminative filter. Let **S**_*b*_, **S**_*w *_denote the between-class and the within-class scatter matrices of EEG segments, respectively.

(A1) $$S_{b}=\sum_{j=1}^{K}n_{j}(M_{j}-M){\left(M_{j}-M\right)}^{\text{T}}$$

(A2) $$S_{w}=\sum_{j=1}^{K}\sum_{i=1}^{n_{j}}(X_{j}(i)-M_{j}){\left(X_{j}(i)-M_{j}\right)}^{\text{T}}$$

where **X**_*j *_(*i*) represents the *i*th EEG segment of class *j*, *K *is the number of classes, *n*_*j *_is the number of EEG segments for class *j*, **M**_*j *_is the average of EEG segments for class *j*, **M **is the average of all the EEG segments. Then the objective function of DSP can be written as [27]:

(A3) $$\max J_{DSP}(W)=\frac{\left|W^{\text{T}}S_{b}W\right|}{\left|W^{\text{T}}S_{w}W\right|}$$

(A3) is in the form of Rayleigh quotient. The solution can be obtained by solving the following generalized eigenvalue problem:

(A4) $$S_{b}w_{q}=\gamma_{q}S_{w}w_{q}$$

where *q *= 1,2,..., *C*, *γ*_*q *_is an eigenvalue and **w**_*q *_is the corresponding eigenvector. Assuming these eigenvalues are sorted in a descending order, only a few eigenvectors W* = [**w**_1_,...,**w**_*d*_] associated with the largest eigenvalues are chosen as the most discriminative spatial filters, where *d *<<*C*. Then each EEG segment is projected as Y* = W*^T ^**X**, Y* ∈ *R*^*d*×*T*^. To obtain the amplitude features of slow potential shifts, we calculate the mean of Y* as $f_{DSP}^{r}=mean(y_{r}^{*})$, where *r *= 1, ..., *d*, $y_{r}^{*}$ is the *r*th row of Y*. In our work, *d *= 2.

For CSP, **W **is optimized to obtain the band power of oscillatory source. It maps EEG segments to the linear subspace where the variance of one class is maximized while the variance of the other class is minimized. The projection vectors achieving the largest and smallest ratios of the variances of the two classes are defined as the most discriminative filters. Assuming **R **denotes the normalized covariance matrix of EEG segment, i.e., **R **= **XX**^T^/trace (**XX**^T^), then the objective function of CSP can be formulated as [28]:

(A5) $$\max J_{CSP}(W)=\frac{\left|W^{\text{T}}R_{1}W\right|}{\left|W^{\text{T}}R_{2}W\right|}$$

where **R**_1 _and **R**_2 _represents the average of the covariance matrices from EEG segments within class 1 and class 2 respectively. Similar to (A3), (A5) is also in the form of Rayleigh quotient. The solution can be obtained by solving the generalized eigenvalue problem:

(A6) $$R_{1}w_{q}=\beta_{q}R_{2}w_{q}$$

where *q *= 1,2,..., *C*, *β*_*q *_is an eigenvalue and **w**_*q *_is the corresponding eigenvector. Suppose these eigenvalues are sorted in a descending order, the eigenvectors associated with the largest and smallest m eigenvalues are chosen as the most discriminative spatial filters, i.e., W* = [**w**_1_,...,**w**_*m*_, **w**_*C*-*m*+1_,...,**w**_*C*_], where *m *<<*C*. Then each EEG segment is projected as Y* = W*^T ^**X**, Y* ∈ *R*^2*m×T*^. To extract the power features, we calculated the logarithm transformation, normalized the variance of Y* by rows $f_{CSP}^{r}=\log\left\{\mathrm{var}(y_{r}^{*})/\sum_{r=1}^{2m}\mathrm{var}(y_{r}^{*})\right\}$. In this paper, *m *= 2. The logarithm transformation is performed to normalize the distribution of the elements in $f_{CSP}^{r}$.

### A3. Relevance of decoding results from different frequency bands

**Figure 9:**
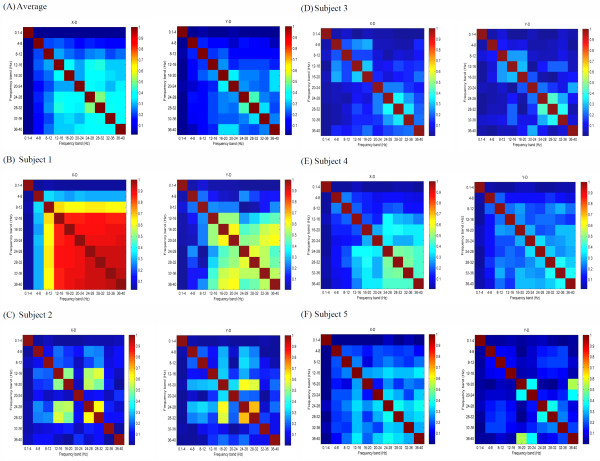
**The absolute correlation coefficient matrices of decoded hand velocities from different frequency bands**.

The absolute correlation coefficient matrices of the decoded hand velocities from different frequency bands are shown in Figure 9. Figure 9(A) illustrates the average of the matrices of the 5 subjects. The decoding result from low frequency band (0.1-4 Hz) is little correlated with those from the frequency bands above 4 Hz in x-dimension and in y-dimension (|cc|<0.05). When we consider the patterns for individual subjects, we obtain similar results as above. Figure 9(B)-(F) show the matrices for the five subjects respectively. For all the 5 subjects, the decoding result from low frequency band (0.1-4 Hz) is not significantly correlated with those from the frequency bands above 4 Hz in x-dimension and in y-dimension (|cc|<0.07, *p *> 0.05 for testing the hypothesis of no correlation).
